# Integrated Analysis of Transcriptome and microRNA Profile Reveals the Toxicity of *Euphorbia* Factors toward Human Colon Adenocarcinoma Cell Line Caco-2

**DOI:** 10.3390/molecules27206931

**Published:** 2022-10-16

**Authors:** Lingyue Zou, Wenqiang Bao, Yadong Gao, Mengting Chen, Yajiao Wu, Shuo Wang, Chutao Li, Jian Zhang, Dongcheng Zhang, Qi Wang, An Zhu

**Affiliations:** 1 Key Laboratory of Ministry of Education for Gastrointestinal Cancer, School of Basic Medical Sciences, Fujian Medical University, Fuzhou 350108, China; 2 Department of Toxicology, School of Public Health, Peking University, Beijing 100191, China; 3 Fujian Provincial Key Laboratory of Zoonosis Research, Fujian Center for Disease Control and Prevention, Fuzhou 350001, China; 4 Key Laboratory of State Administration of Traditional Chinese Medicine for Compatibility Toxicology, Beijing 100191, China; 5 Beijing Key Laboratory of Toxicological Research and Risk Assessment for Food Safety, Beijing 100191, China

**Keywords:** *Euphorbia* semen, *Euphorbia* factors, transcriptome, RNA sequencing, bioinformatics, gene structure

## Abstract

*Euphorbia* factors, lathyrane-type diterpenoids isolated from the medical herb *Euphorbia lathyris* L. (*Euphorbiaceae*), have been associated with intestinal irritation toxicity, but the mechanisms underlying this phenomenon are still unknown. The objective of this study was to evaluate the transcriptome and miRNA profiles of human colon adenocarcinoma Caco-2 cells in response to *Euphorbia* factors L1 (EFL1) and EFL2. Whole transcriptomes of mRNA and microRNA (miRNA) were obtained using second generation high-throughput sequencing technology in response to 200 μM EFL treatment for 72 h, and the differentially expressed genes and metabolism pathway were enriched. Gene structure changes were analyzed by comparing them with reference genome sequences. After 72 h of treatment, 16 miRNAs and 154 mRNAs were differently expressed between the EFL1 group and the control group, and 47 miRNAs and 1101 mRNAs were differentially expressed between the EFL2 group and the control. Using clusters of orthologous protein enrichment, the sequenced mRNAs were shown to be mainly involved in transcription, post-translational modification, protein turnover, chaperones, signal transduction mechanisms, intracellular trafficking, secretion, vesicular transport, and the cytoskeleton. The differentially expressed mRNA functions and pathways were enriched in transmembrane transport, T cell extravasation, the IL-17 signaling pathway, apoptosis, and the cell cycle. The differentially expressed miRNA EFLs caused changes in the structure of the gene, including alternative splicing, insertion and deletion, and single nucleotide polymorphisms. This study reveals the underlying mechanism responsible for the toxicity of EFLs in intestinal cells based on transcriptome and miRNA profiles of gene expression and structure.

## 1. Introduction

*Euphorbia* semen is the ripe and dry seeds of *Euphorbia lathyris* Linnaeus (*Euphorbiaceae*), which are widely distributed in East Asia, South Europe, Russia, Australia, and America [1]. In China, *Euphorbia semen* has been used as a traditional medicine for thousands of years, since the Song dynasty. It is used for the treatment of amenorrhea, edema, abdominal distension, constipation, oliguria, and anuria by oral administration, as well as for therapy for stubborn dermatophytoses and warts by external use on the skin [2,3]. In recent years, *Euphorbia* semen has been reported to have excellent pharmacological actions allowing it to inhibit adipogenesis, reverse tumor multidrug resistance, and treat terminal schistosomiasis, giving it the potential to be developed as a weight-loss and oncotherapy drug [4,5,6].

Safety should be a matter of concern during drug use. In the Compendium of Materia Medica, *Euphorbia* semen was classified as a toxic medicinal herb. In the clinical setting, the recommended oral dosage of *Euphorbia* semen is 1–2 g in accordance with the 2020 edition of the Chinese Pharmacopoeia [7]. At an overdose of 9–15 g, the poisoning symptoms are generally manifested as dizziness, nausea, vomiting, palpitations, spontaneous cold sweating, and a pale complexion. However, blood pressure drops, profuse sweating, cold limbs, shallow and thick breathing, and a weak pulse can occur in serious illness cases [8]. After intragastric administration of an aqueous extract of *Euphorbia* semen, the LD_50_ was 1.7950 g/kg (95% confidence interval of 1.6211–1.9879) in mice [9]. So far, more than 15 types of 240 compounds have been isolated and identified from *Euphorbia* semen, and *Euphorbia* factors belonging to the lathyrane-type diterpenoids family are the main toxic components [10]. *Euphorbia* factors L1 (EFL1) and L2 (EFL2) can accelerate the peristalsis rate of the small intestine and thus contribute to the induction of intestinal tract irritation and diarrhea [11]. The intense intestinal toxicity of *Euphorbia* semen has limited its safe clinical use.

As a classical toxic herb that is used to target to the intestine in traditional Chinese medicine, there is an urgent need to clarify the intestinal toxicological mechanism associated with *Euphorbia* semen, especially regarding *Euphorbia* factors. Next generation high-throughput RNA sequencing (RNA-seq) provides methods with which to find the gene targets of toxic xenobiotics. With the advantages of low cost and time, ultra-high data output and high accuracy, RNA-seq has been introduced into the pharmacological and toxicological studies of traditional medicines such as Fuzi decoction [12], lycorine [13], Shuxinyin formula [14], and so forth. Messenger RNA (mRNA) is formed by transcription and executes its biological functions after being translated into proteins. However, the translation of mRNA into functional proteins can be silenced by microRNA (miRNA) at the post-transcriptional level [15]. miRNA, a kind of small non-coding RNA, is about 21–23 nucleobases in length, and widely exists in viruses, plants, and animals with high evolutionary conservation [16]. miRNA is transcribed from DNA sequences into pri-miRNA, before being processed into pre-miRNA and exported into the cytoplasm. After being processed by RNase III Dicer, the mature miRNA duplex is produced [17]. To form the RNA-induced silencing complex (RISC), the miRNA duplex is loaded into the Argonaut (Ago) family of proteins. The 5′ ends of Ago proteins from the RISCs of miRNA will pair with 3′ untranslated regions (3′-UTRs) of cytoplasmic mRNAs, inhibiting translation or promoting the degradation of the mRNAs [18]. The 3′-UTR is the main region of binding, and other regions of gene promoters, the 5′UTR, and the coding sequence have also been reported as binding locations [19].

To provide evidence for safe clinical drug use, in the present study, we comprehensively used mRNA and miRNA sequencing technologies to explore the mechanisms underlying EFL-induced intestinal toxicity in the human colon adenocarcinoma cell line Caco-2. The gene expression pattern and structural modification were analyzed, and the gene function was enriched by bioinformatics analysis.

## 2. Results

### 2.1. Effects of EFLs on the Survival of Caco-2 Cells

**Figure 1:**
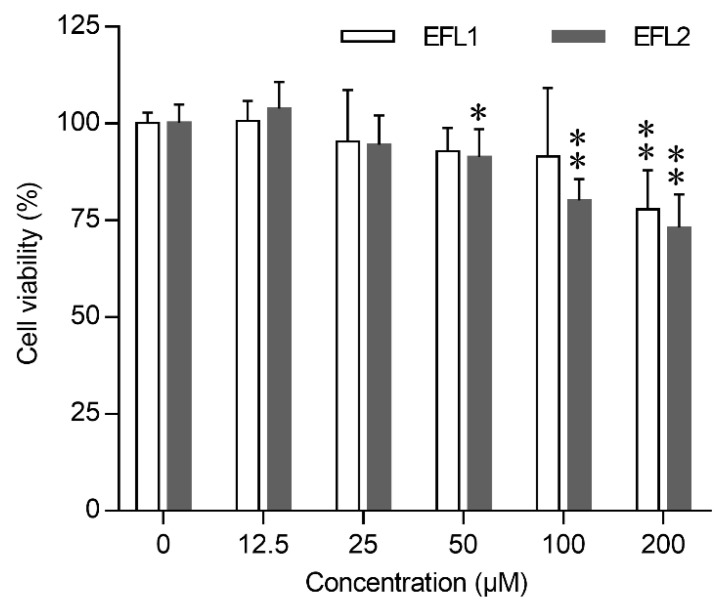
Cytotoxicity of EFLs in Caco-2 cells. Concentration effect of EFLs on cell viability was determined by the MTT assay, the cells viability of each EFLs group was expressed in percentage in comparison with vehicle control group. Data were presented as mean ± standard deviation from three independent experiments. * *p* < 0.05 or ** *p* < 0.01 compared with vehicle control of the same compound.

The cytotoxic effects of EFL1 and EFL2 on Caco-2 were assessed at different time intervals using the MTT assay. EFL1 and EFL2 significantly inhibited cell viability in a concentration-dependent manner (Figure 1). After being exposed to 200 μM EFL1 for 72 h, the cell viability of Caco-2 cells was 77.9%; after being exposed to EFL2 concentrations of 50, 100, and 200 μM, the cell viability decreased to 91.4%, 80.2%, and 73.1%, respectively.

### 2.2. Quality Control and Mapping of RNA Sequencing Data

Based on the cell viability of Caco-2 cells after EFL exposure, a concentration of 200 µM was chosen for further study. High-throughput sequencing was performed for the analysis of mRNA and miRNA expression in Caco-2 cells with or without EFLs exposure. As shown in Table S3, average quantities of 69.4, 74.7, and 71.0 million raw reads of mRNA were obtained from the control, EFL1, and EFL2 groups, respectively. After checking the read quality, the Q20 was 98.3% and Q30 was 94.5% in all samples. In the control, EFL1, and EFL2 groups, 68.4, 73.6, and 70.1 million clean reads were chosen to map to the human reference genome, and the unique mapping ratios were 90.04%, 89.74%, and 89.27%, respectively.

The miRNA quality control is shown in Table S4. The Q20 was 99.43% and Q30 was 98.2% in all the samples. In the control, EFL1, and EFL2 groups, average quantities of 19.3, 18.3, and 18.9 million raw reads were obtained with 16.9, 15.4, and 16.6 million clean reads, respectively, and 8.5, 7.3, and 8.2 million reads were mapped to the positive and negative RNAs of the human reference genome.

### 2.3. Assembly of mRNA Transcripts

The mRNA transcripts were assembled in StringTie software. Based on the network flow algorithms and optional de novo assembly, the complex datasets were assembled into transcripts. The number of transcripts contained in most genes was <4, and the number of genes consisting of only one transcript reached 34,965 (Figure S1A in Supplementary Materials). All samples contained a total of 221,912 transcripts, including 66,911 transcripts > 1800 bp, followed by 49,384 transcripts with a length of 401–600 bp (Figure S1B). Most of the transcripts contained exons with fewer than 10 bp, and the number of transcripts consisting of 2 exons reached 32,846 (Figure S1C).

### 2.4. Functional Annotation of mRNA and miRNA in Databases

The mRNA functional annotation was performed using the COG, GO, KEGG, NR, Swiss-Prot, and Pfam databases (Table S5). In total, 203,268 (97.48%) transcripts and 46,195 (78.45%) genes were successfully annotated. Most of the transcripts and genes were annotated in NR (89.39% and 77.46%, respectively), followed by COG (83.44% and 68.78%, respectively), Swiss-Prot (82.16% and 66.89%, respectively), GO (70.57% and 53.47%, respectively), KEGG (67.14% and 48.15%, respectively), and Pfam (41.17% and 28.11%, respectively).

The known and novel miRNA functional annotation were performed in miRBase and miRDeep2, respectively. An average of 1153, 1109, and 1130 known miRNAs, as well as 356, 355, and 356 novel miRNAs were reported in the control, EFL1, and EFL2 groups, respectively (Table S6).

### 2.5. The Fold Change in mRNA and miRNA Quantified by RT-qPCR

In the differentially expressed genes, a total of 14 mRNAs and 10 miRNAs were detected using RT-qPCR, and the correlation between the fold change in RT-qPCR and mRNA-seq was analyzed using linear regression. In the EFL1 group, the mRNA had a regression equation of Y = 0.892X − 2.761, R^2^ = 0.957 (Figure S2A), with miRNA having Y = 1.108X − 0.025, R^2^ = 0.967 (Figure S2B). In the EFL2 group, the mRNA had a regression equation of Y = 0.924X − 3.759, R^2^ = 0.9903 (Figure S2C), with miRNA having Y = 0.941X + 0.079, R^2^ = 0.9987 (Figure S2D). The gene expression levels detected by RT-qPCR and mRNA-seq were consistent.

### 2.6. Differentially Expressed Genes and miRNA-mRNA Crosstalk

**Figure 2:**
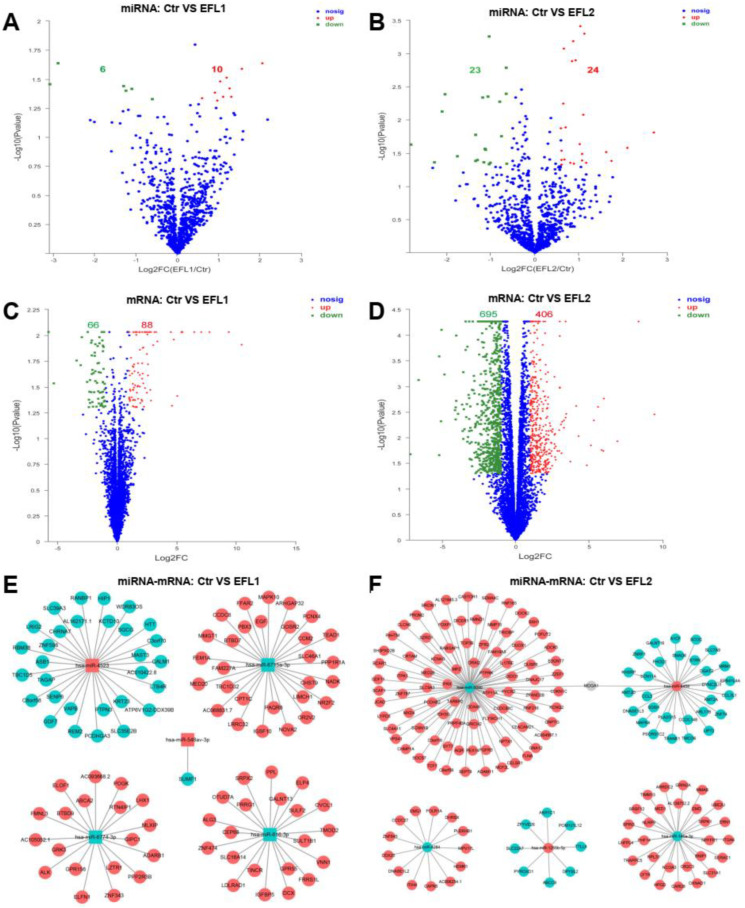
The volcano map of differentially expressed mRNA and miRNA between control and EFLs (**A**–**D**). The crosstalk between top 5 differentially expressed miRNA and corresponding regulated mRNA (**E**,**F**). The square is miRNA, and the circle is mRNA. Red is upregulation, and green is downregulation.

Standards of fold change (FC) ≥ 2 and *p* value ≤ 0.05 were set as significant up- or downregulated differentially expressed genes (DEGs) for mRNA, and FC ≥ 1.5 and *p* value ≤ 0.05 were used for miRNA. After treatment of EFL1, a total of 16 miRNAs were differentially expressed compared with the control group, of which 6 miRNAs were downregulated and 10 miRNAs were upregulated (Figure 2A); 154 mRNAs were differentially expressed, of which 66 mRNAs were downregulated and 88 miRNAs were upregulated (Figure 2C). In the EFL2 group, 47 miRNAs were differentially expressed compared with the control group, of which 23 miRNAs were downregulated and 24 mRNAs were upregulated (Figure 2B); 1101 mRNAs were differentially expressed, of which 695 mRNAs were downregulated and 406 mRNAs were upregulated (Figure 2D).

To describe the miRNA-mRNA crosstalk, the top five differentially expressed miRNAs, namely, hsa-miR-6774-3p, hsa-miR-6715a-3p, hsa-miR-4523, hsa-miR-548av-3p, and hsa-miR-616-3p in the EFL1 group, and hsa-miR-5090, hsa-miR-1255b-5p, hsa-miR-4284, hsa-miR-146a-3p, and hsa-miR-4458 in the EFL2 group were selected. As shown in Figure 2E,F, the target genes of miRNA are presented in respect of three databases: miRnada, TargetScan, and RNAhybrid.

The overlaps between the target mRNAs of the top five differentially expressed miRNAs and differentially expressed mRNAs were analyzed. In the EFL1 group, 100 target mRNAs of miRNAs were reported, but no genes were in the overlap. In the EFL2 group, 162 target mRNAs of miRNAs were reported, and 7 of them were in the overlap, namely MDGA1, C3orf18, QRICH2, PROM2, AKR1C1, HPGD, and CCDC148.

### 2.7. Functional Annotation of Differentially Expressed mRNA in EFL-Treated Cells

**Figure 3:**
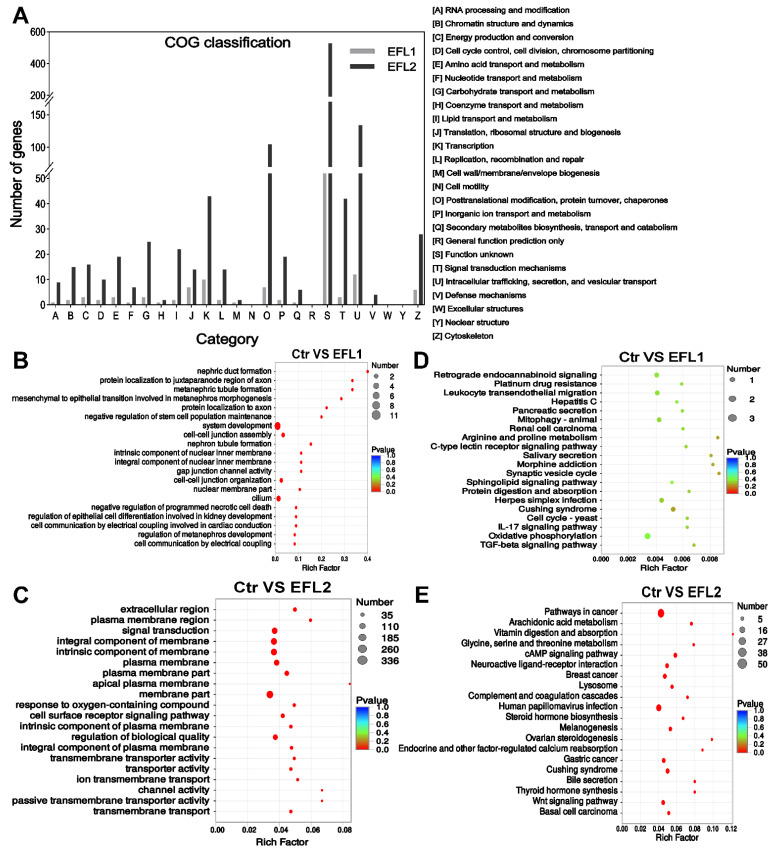
(**A**) The COG classification of differentially expressed mRNA in EFL-treated Caco-2 cells. (**B**) GO and (**C**) KEGG annotation of the differentially expressed mRNA in the EFL1 group. (**D**) GO and (**E**) KEGG annotation of the differentially expressed mRNA in the EFL2 group.

The COG functional annotation of differentially expressed mRNA is shown in Figure 3A. Most mRNA functions were enriched in intracellular trafficking, secretion, and vesicular transport, as well as post-translational modification, protein turnover, chaperones, transcription, signal transduction mechanisms, and the cytoskeleton.

The GO enrichment of the differentially expressed mRNA indicated that EFL1 affects the negative regulation of programmed necrotic cell death, cell–cell junction assembly, gap junction channel activity, cell–cell organization, and cell communication by electrical coupling (Figure 3B). EFL2 affects the response to oxygen-containing compounds, the cell surface receptor signaling pathway, transmembrane transporter activity, ion transmembrane transport, channel activity, and passive transmembrane transporter activity (Figure 3D).

The KEGG enrichment of the differentially expressed mRNA indicated that EFL1 mainly affects mitophagy, protein digestion and absorption, the IL-17 signaling pathway, oxidative phosphorylation, and the TGF-beta signaling pathway (Figure 3C). EFL2 affects the arachidonic acid metabolism, cAMP signaling pathway, lysosome, bile secretion, and the Wnt signaling pathway (Figure 3E).

### 2.8. Functional Annotation of the Target Genes of Differentially Expressed miRNA in EFL-Treated Cells

**Figure 4:**
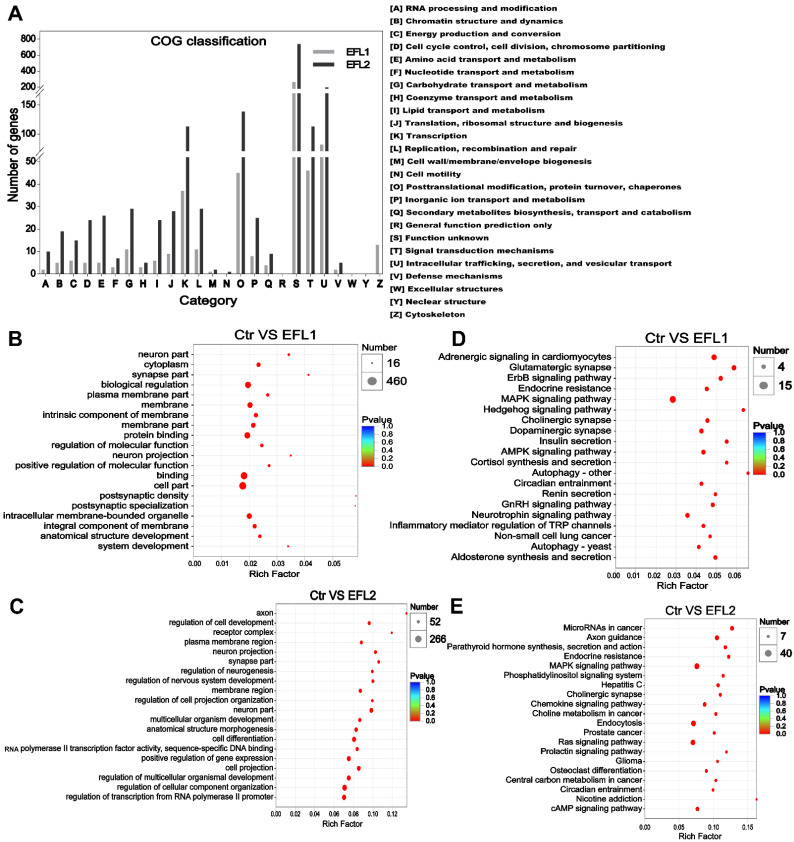
(**A**) The COG classification of differentially expressed miRNA in EFL-treated Caco-2 cells. (**B**) GO and (**C**) KEGG annotation of the differentially expressed miRNA in the EFL1 group. (**D**) GO and (**E**) KEGG annotation of the differentially expressed miRNA in the EFL2 group.

The COG functional annotation of the target genes of differentially expressed miRNAs is shown in Figure 4A, most functions were enriched in terms of transcription, post-translational modification, protein turnover, chaperones, signal transduction mechanisms, intracellular trafficking, secretion, and vesicular transport.

In the EFL1-treated group, the GO enrichment of the target genes of differentially expressed miRNAs showed enrichment of the term’s biological regulation, membrane, protein binding, and intracellular membrane-bounded organelle (Figure 4B). In the EFL2-treated group, the enriched terms were regulation of multicellular organismal development, cell projection, regulation of transcription from the RNA polymerase II promoter, positive regulation of gene expression and membrane region (Figure 4D).

In the EFL1-treated group, the KEGG enrichment of the target genes of differentially expressed miRNA showed enrichment of the terms AMPK signaling pathway, autophagy, and inflammatory mediator regulation of TRP channels (Figure 4C). In the EFL2-treated group, the enriched terms were the MAPK signaling pathway, chemokine signaling pathway, Ras signaling pathway, and cAMP signaling pathway (Figure 4E).

### 2.9. The Protein–Protein Interaction (PPI) of Differentially Expressed mRNA

**Figure 5:**
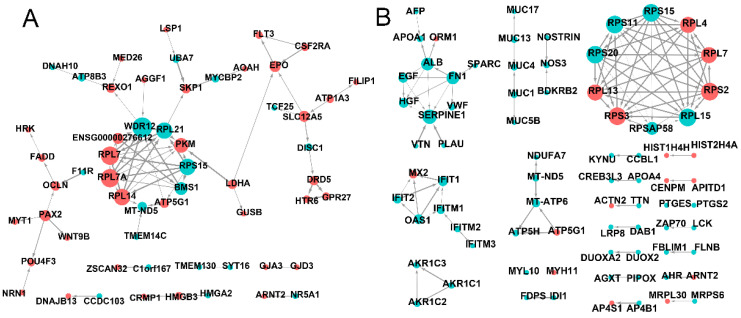
The PPI network of differentially expressed mRNA in EFL1 (**A**) and EFL2 (**B**) treated Caco-2 cells.

The genes network was investigated through analysis of PPIs. After EFL1 treatment for 72 h in Caco-2 cells, the core genes involved in the network were shown to be *RPL7*, *RPL7A*, *RPL14*, *MT-ND5*, *ATP5G1*, *BMS1*, *RPS15*, *PKM*, *RPL21*, *WDR12*, *SKP1*, *EPO*, *SLC12A5*, *OCLN* and *PAX2* (Figure 5A). These genes regulate peptide chain elongation, translational control, respiratory electron transport, ATP synthesis by chemiosmotic coupling, rRNA processing in the nucleus and cytosol, RNA binding, processing of capped intron-containing pre-mRNA, mitotic G1 phase and G1/S transition, development EPO-induced Jak-STAT pathway, K-Cl cotransporter activity, tight junction paracellular permeability barrier, and transcription factor binding.

In EFL2-treated Caco-2 cells, the core genes involved in the PPI network were shown to be *RPS15*, *RPL4*, *RPL7*, *RPS2*, *RPL15*, *RPSAP58*, *RPS3*, *RPL13*, *RPS20*, *RPS11*, *ALB*, *FN1*, *SERPINE1*, *IFIT1*, *IFITM1* and *MT-ATP6* (Figure 5B). These genes were found to be involved in peptide chain elongation, RNA binding and structural constituents of the ribosome, laminin receptor activity, the response to elevated platelet cytosolic Ca^2+^, integrin family cell surface interactions, signaling receptor binding, the interferons-mediated signaling pathway, the innate immune system, respiratory electron transport, ATP synthesis by chemiosmotic coupling, and transcriptional activation of mitochondrial biogenesis.

### 2.10. The Functional Annotation of Differentially Co-Expressed mRNA

**Figure 6:**
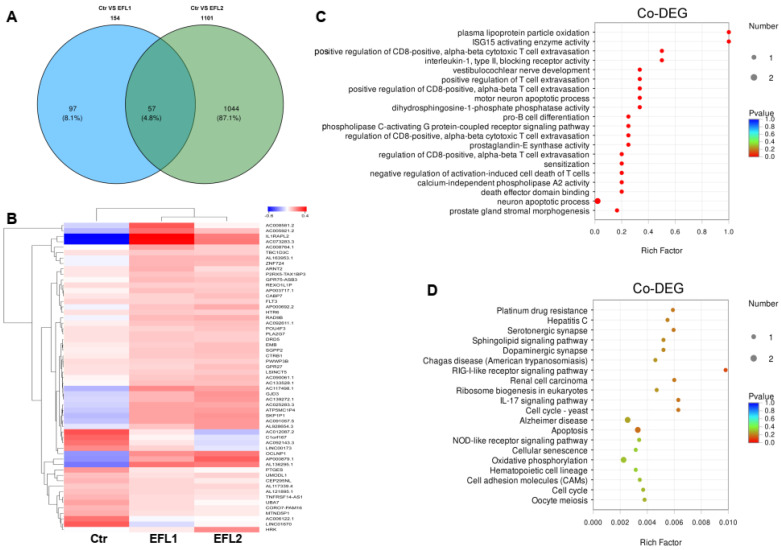
(**A**) The Venn diagram of differentially expressed mRNA in each EFL-treated group. The cluster analysis heat map (**B**), GO (**C**) and KEGG (**D**) enrichment of 57 differentially co-expressed mRNAs.

After EFL1 and EFL2 treatment in Caco-2 cells, 57 mRNAs were the differentially co-expressed as the union (Figure 6A), and a cluster analysis heat map was created (Figure 6B). The GO enrichment indicated that the differentially co-expressed mRNA affected plasma lipoprotein particle oxidation, the positive regulation of T cell extravasation, death effector domain binding, and the neuron apoptotic process (Figure 6C). The KEGG enrichment terms included platinum drug resistance, the RIG-I-like receptor signaling pathway, ribosome biogenesis in eukaryotes, IL-17 signaling pathway, apoptosis, the NOD-like receptor signaling pathway, oxidative phosphorylation, cell adhesion molecules, and the cell cycle (Figure 6D).

### 2.11. Visual Analysis of Metabolic Pathways Involved in Differentially Expressed Genes

After the treatment of Caco-2 cells for 72 h, EFL1 affected a few metabolic pathways including nucleotide metabolism, the metabolism of cofactors and vitamins, xenobiotic biodegradation and metabolism (glyoxylate and dicarboxylate metabolism, citrate cycle, chlorocyclohexane and chlorobenzene degradation, and oxidative phosphorylation), carbohydrate metabolism (pyruvate metabolism), and amino acid metabolism (phosphonate and phosphinate metabolism, and D-Alanine metabolism) (Figure S3A). EFL2 affected most of the metabolic pathways, including the metabolism of terpenoids and polyketides, xenobiotic biodegradation and metabolism, amino acid metabolism, nucleotide metabolism, the metabolism of cofactors and vitamins, the metabolism of other amino acids, and the biosynthesis of other secondary metabolites, especially glycan biosynthesis and metabolism, lipid metabolism, carbohydrate metabolism, and energy metabolism (Figure S3B).

### 2.12. Relative Expression Levels of Proteins Involves in Oxidative Stress and Mitochondria

**Figure 7:**
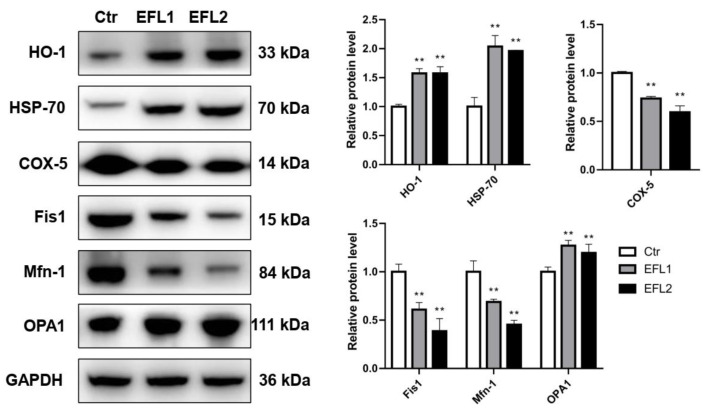
The relative expression levels of proteins involved in oxidative stress and mitochondrial energy metabolism. ** *p* < 0.01 compared with vehicle control group.

In the PPI network (Figure 5), genes involved in oxidative stress and mitochondrial energy metabolism were presented. In the Western blotting assay, heme oxygenase 1 (HO-1) and heat shock 70 kDa protein (HSP-70) were selected as the biomarkers of oxidative stress, cytochrome c oxidase 5 (COX-5), mitochondrial fission 1 protein (Fis1), mitofusin-1 (Mfn-1), and dynamin-like 120 kDa protein optic atrophy 1 (OPA1) were the biomarkers of mitochondrial energy metabolism. As shown in Figure 7, after treatment of EFLs, HO-1 and HSP-70 were activated, and the COX-5, Fis-1, and Mfn-1 were inhibited, as well as OPA-1 was increased.

### 2.13. Gene Structure Change after EFL Treatment in Caco-2 Cells

In the Caco-2 cells treated with EFLs for 72 h, 2022 and 5385 AS were reported in the EFL1 and EFL2 groups, respectively, and most of them were SEs, with a total of 1499 (74.1%) and 3830 (71.1%), respectively (Table S7).

The Indel numbers of 17 regions were recorded. The totals of 3240.3, 3157.3, and 2988.7 Indels were found in the control, EFL1, and EFL2 groups, respectively, and the number in the EFL2 group was significantly decreased when compared with the control group (Table S8). Most of the Indels were distributed in intronic, UTR3, ncRNA_intronic, intergenic, and ncRNA_exoinc regions, and those with no Indel were distributed in the exonic-splicing and ncRNA_splicing regions.

As shown in Table S9, 564,449, 562,139, and 509,564 SNP sites were recorded in the control, EFL1, and EFL2 groups, respectively. The total numbers of SNP, C/T and G/A transition, and T/A transversion sites in the EFL2 group were significantly decreased when compared with the control group. The SNP sites were distributed in all of the 17 regions, mainly in the intronic, UTR3, intergenic, exonic, and ncRNA_exonic regions (Table S10). No regional distributions were significantly different.

## 3. Materials and Methods

### 3.1. Materials

**Figure 8:**
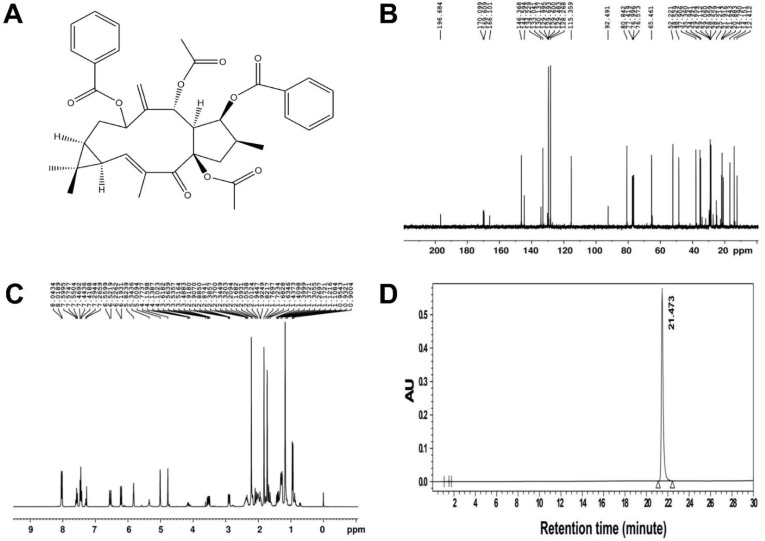
(**A**) The chemical structure of EFL2. The nuclear magnetic resonance spectra of EFL2. The left panel was the ^13^C spectra (**B**), and the right panel was the ^1^H spectra (**C**). The purity of EFL2 detected by HPLC (**D**). Chromatographic conditions: Gemini C18 column (250 × 4.6 mm, 4 μm particle size); mobile phase of methanol:water = 65:35; flow rate: 1.0 mL/min; detection wavelength: 250 nm; column temperature: 35 °C; injection volume: 5 μL; injection concentration: 2 mg/mL in methanol. The retention time of EFL1 was 21.473 min, and the area percent of EFL1 was 99.63%.

EFL1 and EFL2 were purchased from Spring & Autumn Biological Engineering Co., Ltd. (Nanjing, China). The chemical structure and purity of EFL1 were described in our previous study [20]. The chemical structure of EFL2 was confirmed by 400 MHz ^13^C and ^1^H nuclear magnetic resonance (NMR) spectra (Bruker, Rheinstetten, Germany), and the purity of EFL2 was 99.63% as determined by high-performance liquid chromatography (HPLC) (Agilent, Santa Clara, CA, USA) (Figure 8).

### 3.2. Cell Culture and Treatment

Caco-2 cells were obtained from Biotides Biotechnology Co., Ltd. (Beijing, China), and the genetic information was identified by short tandem repeats. The cells were cultured in minimum essential medium (MEM, Gibco, New York, NY, USA) supplemented with 10% fetal bovine serum (Gibco), 100 U/mL penicillin G sodium salt, 100 μg/mL streptomycin sulfate, 1% sodium pyruvate, 1% GlutaMAX and 1% non-essential amino acid. Cells were maintained in an incubator (Thermo Fisher, Langenselbold, Germany) in an atmosphere of 5% CO_2_ at 37 °C. EFLs were dissolved in dimethyl sulfoxide (DMSO) and then diluted in MEM medium to concentrations of 12.5, 25, 50, 100, and 200 μM. The final working concentration of DMSO in cell culture experiments did not exceed 1%.

### 3.3. Measurement of Cytotoxicity Using the MTT Assay

The viability of Caco-2 cells exposed to EFLs was evaluated using MTT cytotoxicity assay. In brief, 10^4^ cells were seeded in 96-well plates and treated with gradient concentrations of EFLs for 72 h. After incubation, cells received 20 μL 5 mg/mL MTT per well and were maintained at 37 °C for 4 h. After removing the medium, 100 μL of 4% isopropanol acid was added to dissolve the intracellular formazan product. The optical density of formazan was measured at a wavelength of 570 nm using a microplate reader (Omega, Ortenberg, Germany).

### 3.4. RNA Extraction and cDNA Library Construction

Caco-2 cells were seeded in 25 cm^2^ flasks, and the total RNA was isolated by adding 500 μL TRIzol (Invitrogen, Carlsbad, CA, USA). The RNA quality was determined using 1% agarose gel electrophoresis. RNA samples with a qualified purity of OD_260_/OD_280_ ≥ 1.8 and OD_260_/OD_230_ ≥ 1.5 were measured using Nanodrop 2000 (Thermo Scientific). The RNA integrity number (RIN) was further assessed using a bioanalyzer 2100 (Agilent).

Poly-(A)-containing mRNA was isolated using oligo (dT) magnetic beads. A fragmentation buffer was added to disrupt mRNA into 300 bp short fragments, and these were then used as templates to synthesize the first-strand cDNA using reverse transcriptase and random hexamers. Second-strand cDNA was synthesized using dNTPs, RNase H, and DNA polymerase I. The double-strand cDNA was subjected to end repair and adapter ligation. Adapter-modified fragments were purified and amplified to create a cDNA library.

The Truseq Small RNA kit (Illumina, San Diego, CA, USA) was used to construct the cDNA library of miRNA. Briefly, 1 μg total RNA was used to add 3′ and 5′ adaptors, for the synthesis of first-strand cDNA by random hexamers. The cDNA was amplified by 12 PCR cycles and purified in 6% Novex TBE PAGE gel, before being quantified by PicoGreen TBS380. After bridge PCR by cBot, a cluster was generated for subsequent sequencing. Assays were performed in triplicate.

### 3.5. Transcriptome Sequencing and Bioinformatics Analysis

Transcriptome sequencing was performed on the second-generation high-throughput sequencing platform of Novaseq 6000 (Illumina). Homo sapiens GRCh38 was applied as the reference genome. The FASTX-Toolkit 0.0.14 was used to calculate the base mass distribution and base error rate distribution. The raw reads were subjected to adapter trimming, and low-quality reads were filtered using SeqPrep and Sickle [21]. The high-quality clean reads were aligned to the human genome using TopHat2 [22]. The transcript assembly was performed in StringTie. The overlapping read pairs were assembled into super reads. Then, the super reads were aligned to the reference genome to construct the graph of the splice site. The paths with high read coverage were retained and assembled to form transcripts. Then, they were annotated in the Clusters of Orthologous Groups (COG), Gene Ontology (GO), Kyoto Encyclopedia of Genes and Genomes (KEGG), Non-Redundant Protein Sequence Database (NR), Swiss-Prot and Pfam databases for mRNA using DIAMOND [23], Blast2GO [24], HMMER [25] and KOBAS2.1 [26] software and servers, and miRBase [27] or miRDeep2 [28] were used for miRNA annotation. The gene expression levels were calculated based on transcripts per million reads (TPM) using RSEM [29]. iPath3.0 (http://pathways.embl.de, accessed on 28 July 2019) was used to visualize and analyze metabolic pathways [30]. It contains three types of pathway maps: the metabolic pathways including 146 KEGG pathways involved in the overall metabolism of biological systems, the regulatory pathways including 22 KEGG regulatory pathways, and the biosynthesis of secondary metabolites including 58 KEGG pathways.

### 3.6. miRNA-mRNA Network

The target gene of miRNA was analyzed by the intersection of three databases: miRnada [31], TargetScan [32], and RNAhybrid [33]. In the paradigm suggesting that miRNAs are restrictive to mRNA, miRNA can couple with mRNA to silence or degrade it. Furthermore, apart from the classic negative regulation, a positive correlation of mRNA and miRNA has also been reported as the complementary mechanism and perturbation of feedback loops. This positive regulation was not included in the present study. Finally, Cytoscape 3.5.1 was used to construct the protein–protein interaction between miRNA and mRNA.

### 3.7. RNA Isolation and Quantitative Real-Time Polymerase Chain Reaction (RT-qPCR)

RT-qPCR was performed to identify the expression of DEGs. Total RNA from Caco-2 cells was isolated using TRIzol reagent (Invitrogen, Carlsbad, CA, USA). To quantify the relative gene expression, the primers were synthesized, as shown in Tables S1 and S2, with β-actin and U6 used as the endogenous controls of mRNA and miRNA, respectively. The universal PCR reverse primer and U6 forward primer were used for the cDNA reverse transcription of miRNA. RT-qPCR was performed using a 20 μL reaction volume. The relative gene expression was calculated using the 2^−ΔΔCt^ method (Schmittgen and Livak, 2008), and the fold change was normalized to that observed in the control group.

### 3.8. mRNA Gene Structure Analysis

The gene structure changes were identified by aligning sequenced sequences with reference genome sequences. Single nucleotide polymorphisms (SNPs) are genetic markers on the genome formed by single nucleotide variations. Another structure change is the insertion and deletion (InDel) of fragments relative to the reference genome, which may contain one or more bases. The SNP types, including base conversion and transversion, mutation frequency and depth, as well as the Indel frequency and depth, were calculated using Samtools software [34]. The gene sequence of eukaryotic cells contains introns and exons. After the gene is transcribed into mRNA precursors, the introns are removed by RNA splicing bodies, while the exons are retained in the mature mRNA. Alternative splicing (AS) means that unspliced RNA can have multiple forms of exon splicing, so that a gene can translate different proteins, thereby improving the complexity and adaptability of the system under various physiological conditions. The AS, including the skipped exon (SE), alternative 5′ splice site (A5SS), alternative 3′ splice site (A3SS), mutually exclusive exon (MXE), and retained intron (RI), were quantified and analyzed in the Junction Count Only mode of rMATS software [35].

### 3.9. Protein Expression Detected by Western Blotting

The cells were cultured in 6-well plates and treated with 200 µM EFL1 or EFL2 for 72 h. Cells were lysed by RIPA and the protein concentrations were quantified by BCA method. Equal amounts of 20 μg proteins were loaded for separation by SDS-page electrophoresis, and then electrophoretically transferred to polyvinylidene difluoride membranes. After blocking in 5% skim milk and successively incubation with primary and secondary antibodies (Proteintech and Abclonal, Wuhan, China), the membranes were exposed to ECL chemiluminescence solution (Beyotime, Shanghai, China) for imaging.

### 3.10. Statistical Analysis

The data are expressed as the mean ± standard deviation. One-way analysis of variance was performed using SPSS software (IBM, Armonk, NY, USA) to compare the differences between groups. Values of * *p* < 0.05 or ** *p* < 0.01 were considered statistically significant.

## 4. Discussion

*Euphorbiaceae* plants are mainly distributed in the tropics and subtropics and include more than 300 genera and more than 8000 species. In China, there are more than 80 plants from the *Euphorbia* genus, and 40 of them are used for traditional medicines, while over 40% of them are toxic [36]. *Euphorbia kansui* S.L.Liou ex S.B.Ho [37], *Euphorbia pekinensis* Rupr. [38], *Euphorbia ebracteolata* Hayata [39], *Euphorbia fischeriana* Steud. [40], *Euphorbia lathyris* L., and *Euphorbia hirta* L. [41], have been reported to be poisonous. There are typical gastrointestinal irritation xenobiotic substances that induce clinical symptoms of acute gastroenteritis, nausea, vomiting, abdominal pain, diarrhea and hematochezia. Although the toxicity effects have been reported in many clinical cases, the toxicity mechanism has still not been fully revealed.

In the 2020 edition of the Chinese Pharmacopoeia, 83 traditional Chinese medicinal materials are considered to be toxic. Depending on the intensity of toxicity, these materials are categorized into three levels, and *Euphorbia* semen belongs to the middle level [42]. The excellent pharmacological efficacy of *Euphorbia* semen makes it difficult to remove from the list of medicinal plants, but its clinical use is severely restricted due to the drug’s safety. In a pharmacokinetic study, SD rats and rhesus macaques were used for the intragastric administration (i.g.) and intravenous injection of EFL1, and the T_1/2_ (i.g.) values were 12.6 h and 9.9 h, while the bioavailability values were 2.0 and 4.6, respectively [43]. Although the oral bioavailability of EFL1 was low, toxicological studies has reported that the methanol, ethyl acetate, petroleum ether, and aqueous extract of *Euphorbia* semen cause reduced spontaneous activities, watery stools, wet perioral hair, urinary incontinence, and tics in mice treated with EFL1 (i.g.) once [44,45]. Jaretzky and Köhler used 2.5 mL/100 g seed oil of *Euphorbia* semen for once i.g. dose in rats. Diarrhea was reported as the toxicity endpoint, and this was related to accelerated intestinal rhythm movement [11]. In our previous study, EFL1 impaired the intestinal barrier integrity and accelerated the defecation cycle in the coelomic model organism *Caenorhabditis elegans* [46].

The main toxic component of *Euphorbia* semen is lathyrane, of which EFL1 and EFL2 have high proportions of the mass fraction [47,48]. In the present study, these two toxic compounds were selected to explore the toxicological mechanism of *Euphorbia* semen, and the transcriptome profiles were analyzed based on the Caco-2 intestinal cell line model. After treatment of EFLs for 72 h, the cell viability was inhibited. Through second-generation high-throughput sequencing of mRNA and miRNA, more differentially expressed genes were discovered in the EFL2 group, suggesting that EFL2 is more toxic to Caco-2 cells. Through gene assembly and functional annotation, gene function enrichment was performed. A previous study found that EFL1 inhibited the mRNA expression of aquaporin, a protein facilitating water transport across the plasma membrane, thus contributing to abnormal intestinal permeability [49]. In our study, GO and KEGG enrichment of EFL1 found enrichment of the terms cell–cell junction, transporter activity and transmembrane transport. This factor was also shown to regulate ion and molecule transport and result in osmolality disturbance. The results were consistent with previous reports in the literature. The gene function enrichment in the EFL2 group included the terms cAMP, IL-17, and TGF-beta signaling pathway, suggesting that ATP and energy metabolism dysfunction as well as the inflammatory response occur in intestinal cells. Once the cells are in a state of energy deficiency, the reverse concentration transport will be blocked, resulting in abnormal intestinal osmotic pressure [50]. The inflammation usually damages the integrity of the intestinal barrier, and the tight junctions and adherent junctions between cells break, allowing the intestinal endotoxins produced by the intestinal flora to be transported to the circulatory system [51]. Intestinal inflammation is the leading cause of diarrhea [52].

Next-generation high-throughput sequencing methods have been applied in the study of transcriptomes and have revolutionized the understanding of gene structure changes in AS, Indel and SNP, the most abundant gene polymorphisms in eukaryotic genomes that are usually associated with various diseases [53]. Nearly all transcripts from human protein-coding genes undergo several forms of AS, such as SE, A5SS, A3SS, MXE, and RI. After the gene is transcribed into mRNA precursors, the introns are moved by RNA splicing bodies, while the exons are retained in the mature mRNA. Unspliced RNA has multiple forms of exon splicing, so a gene can translate different proteins at different times and in different environments, thereby increasing the complexity or adaptability of the organism under different physiological conditions [54]. An SNP refers to a genetic marker formed by a single nucleotide variation on the genome. SNPs are numerous in number and rich in polymorphisms. SNPs appear most frequently on CG sequences, and most of them are C/T conversions, since the C in CG is often methylated, and becomes thymine after spontaneous deamination. It is divided into conversion and transversion, according to the characteristics of the SNP [55]. The Indel refers to the difference in the whole genome between two parents, where one parent has a certain number of nucleotide insertions or deletions in the genome relative to the other parent. It involves the insertion or deletion sequence of small fragments in the genome, the lengths of which are between 1 and 50 bp [56]. Indel occurrence is mainly related to genomic features and DNA replication errors. It has an average density of one Indel per 7.2 kb in the human genome [57]. In the present study, after treatment of EFL1 or EFL2 in Caco-2 cells, thousands of AS sites were discovered, and most of them were SEs. This might be the adaptive response for cells. The number of Indels in the EFL2 groups was significantly decreased, suggesting the less genetic length polymorphism. The C/T and G/A transitions, T/A transversion, and total SNP numbers in EFL2-treated Caco-2 cells were decreased, and SNPs occurring in any region of the gene had the potential to affect the protein structure or expression levels of the gene products. The EFLs, especially EFL2, could induce changes in the AS, Indel, and SNP sites in Caco-2 cells and thus contribute to gene expression and affect the biological function.

## 5. Conclusions

In conclusion, as the main diarrheal toxicity components of *Euphorbia* semen, EFL1 and EFL2 caused profiles changes in miRNA and mRNA, and the gene functions were enriched in the terms transmembrane transport, T cell extravasation, the IL-17 signaling pathway, apoptosis, and the cell cycle. The hsa-miR-6774-3p and hsa-miR-5090 were identified as the key miRNAs for the silencing and degradation of target mRNAs after the exposure of EFLs. Regarding the gene structure, EFL2 induced the AS, Indel and SNP components. This study provides gene expression, functional and structural information regarding Caco-2 cells exposed to EFLs. To the main deficit of the present study, all the conclusions were conducted based on bioinformatic analysis, and further molecular biological experiments should be performed to validate whether the DEGs-related functions or pathways were changed in reality.

## Data Availability

All data are included in this manuscript.

## Supplementary Materials

The following supporting information can be downloaded at: https://www.mdpi.com/article/10.3390/molecules27206931/s1, Figure S1: Assembly of mRNA transcripts by StringTie. (A) Transcripts per gene, (B) length distribution of transcripts, and (C) exons per transcript.; Figure S2: The correlation between the fold change of RT-qPCR and mRNA-seq of part of differentially expressed mRNA and miRNA in EFL1 group (A and B) and EFL2 group (C and D).; Figure S3. The visual analysis of metabolic pathways involved in differentially expressed genes after (A) EFL1 or (B) EFL2 treatment in Caco-2 cells.; Table S1. Primer sequences for miRNA of Caco-2 cells; Table S2. Gene descriptions and sequences of qRT-PCR primers for mRNA of Caco-2 cells; Table S3. Summary of reads and mapping rate of mRNA sequences of Caco-2 cells with or without EFLs treatment; Table S4. Summary of reads and mapping rate of miRNA sequences of Caco-2 cells with or without EFLs treatment; Table S5. Functional annotation of transcriptome data in six public databases; Table S6. The miRNA numbers including known and novel miRNAs in each sample; Table S7. The AS number in Caco-2 cells treated with EFLs; Table S8. The Indel regions distribution after EFLs treatment in Caco-2 cells; Table S9. The SNP number in Caco-2 cells treated with EFLs; Table S10. The SNP regions distribution after EFLs treatment in Caco-2 cells.

## Sample Availability

Samples of the compounds are not available from the authors.

## Abbreviations

3′-UTR	3′ untranslated region
A3SS	Alternative 3′ splice site
A5SS	Alternative 5′ splice site
Ago	Agronaut
AS	Alternative splicing
COG	Clusters of orthologous groups of proteins
COX-5	Cytochrome c oxidase 5
DEG	Differentially expression genes
DMSO	Dimethyl sulfoxide
EFL1	*Euphorbia* factor L1
EFL2	*Euphorbia* factor L2
Fis1	Mitochondrial fission 1 protein
GO	Gene ontology
HO-1	Heme oxygenase 1
HPLC	High performance liquid chromatography
HSP-70	Heat shock 70 kda protein
InDel	Insertion and deletion
KEGG	Kyoto Encyclopedia of Genes and Genomes
Mfn-1	Mitofusin-1
miRNA	Microrna
mRNA	Messenger RNA
MXE	Mutually exclusion exon
NMR	Nuclear magnetic resonance
OPA1	Optic atrophy 1
RI	Retained intron
RIN	RNA integrity number
RISC	RNA-induced silencing complex
RNA-seq	RNA sequencing
RT-qPCR	Quantitative real-time polymerase chain reaction
SE	Skipped exon
SNP	Single nucleotide polymorphisms
