# Supplementary material for: Integrated Analysis of Transcriptome and microRNA Profile Reveals the Toxicity of Euphorbia Factors toward Human Colon Adenocarcinoma Cell Line Caco-2
Source: Molecules. 2022 Oct 16;27(20):6931. doi: 10.3390/molecules27206931 (PMC9608949; doi:10.3390/molecules27206931)
Supplement: Supplementary file 1 [file molecules-27-06931-s001.zip › molecules-1952634-supplementary.pdf]

Supplementary Material

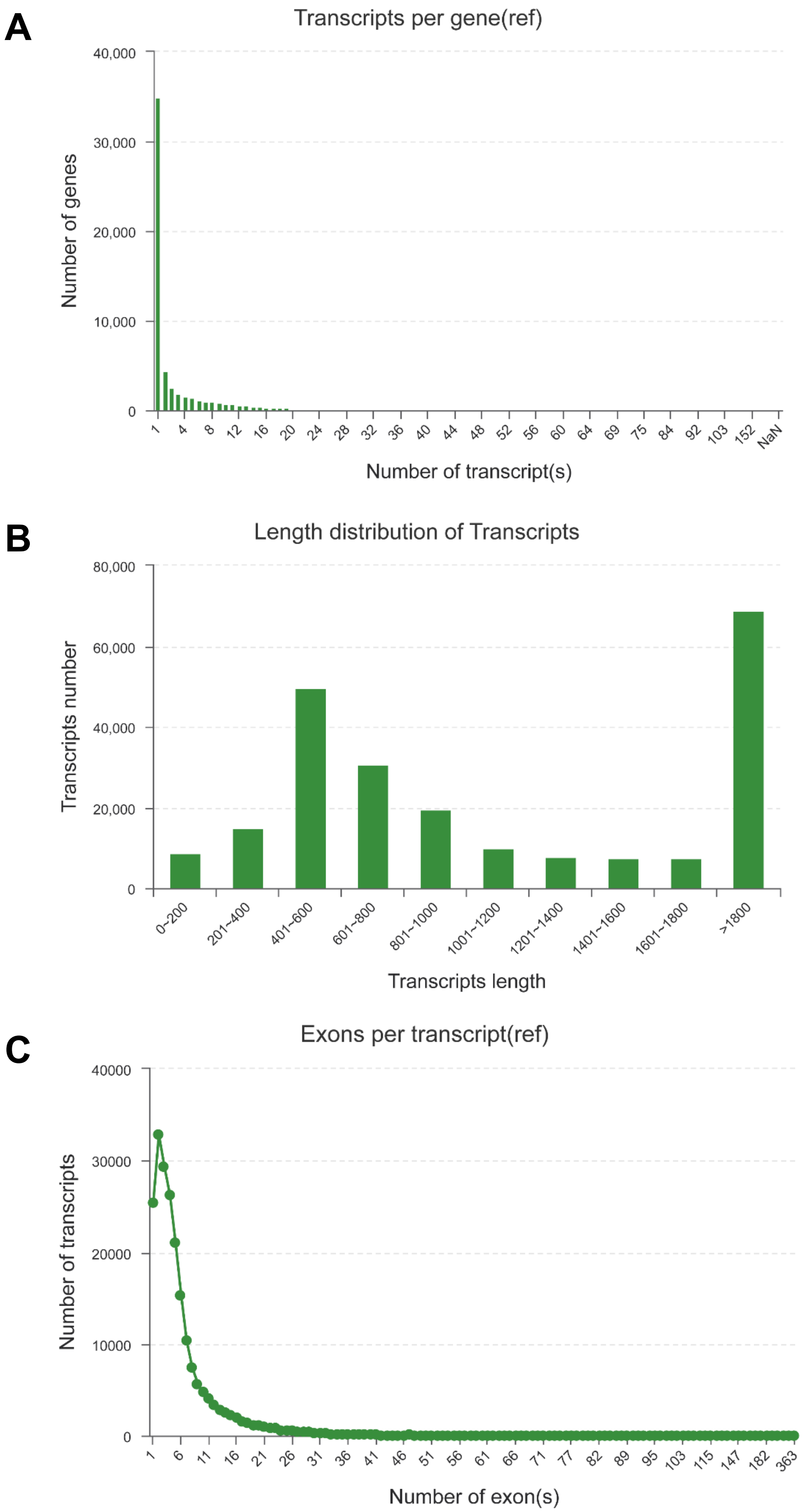

Figure S1. Assembly of mRNA transcripts by StringTie. (A) Transcripts per gene, (B) length distribution of transcripts, and (C) exons per transcript.

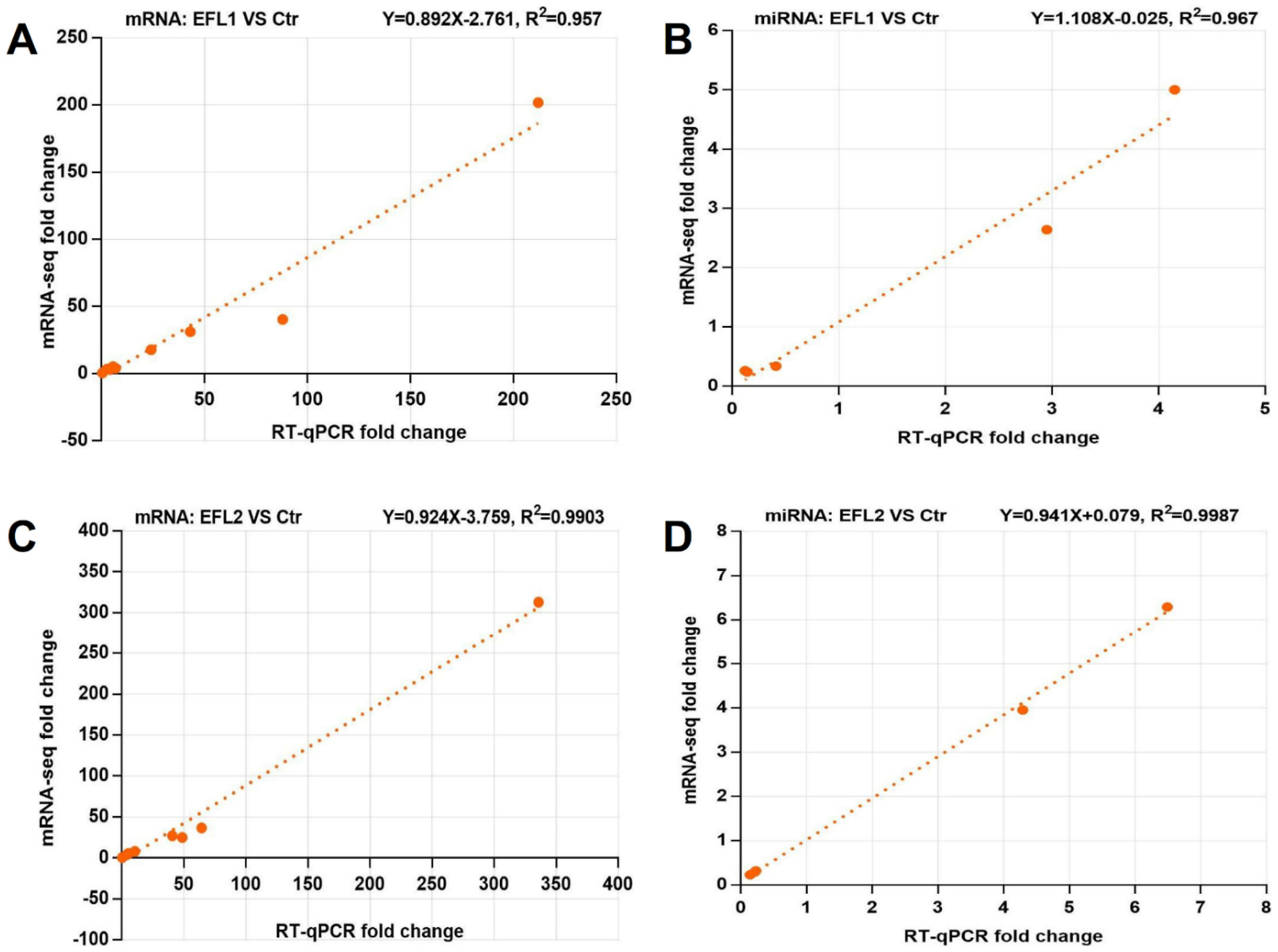

Figure S2. The correlation between the fold change of RT-qPCR and mRNA-seq of part of differentially expressed mRNA and miRNA in EFL1 group (A and B) and EFL2 group (C and D).

A

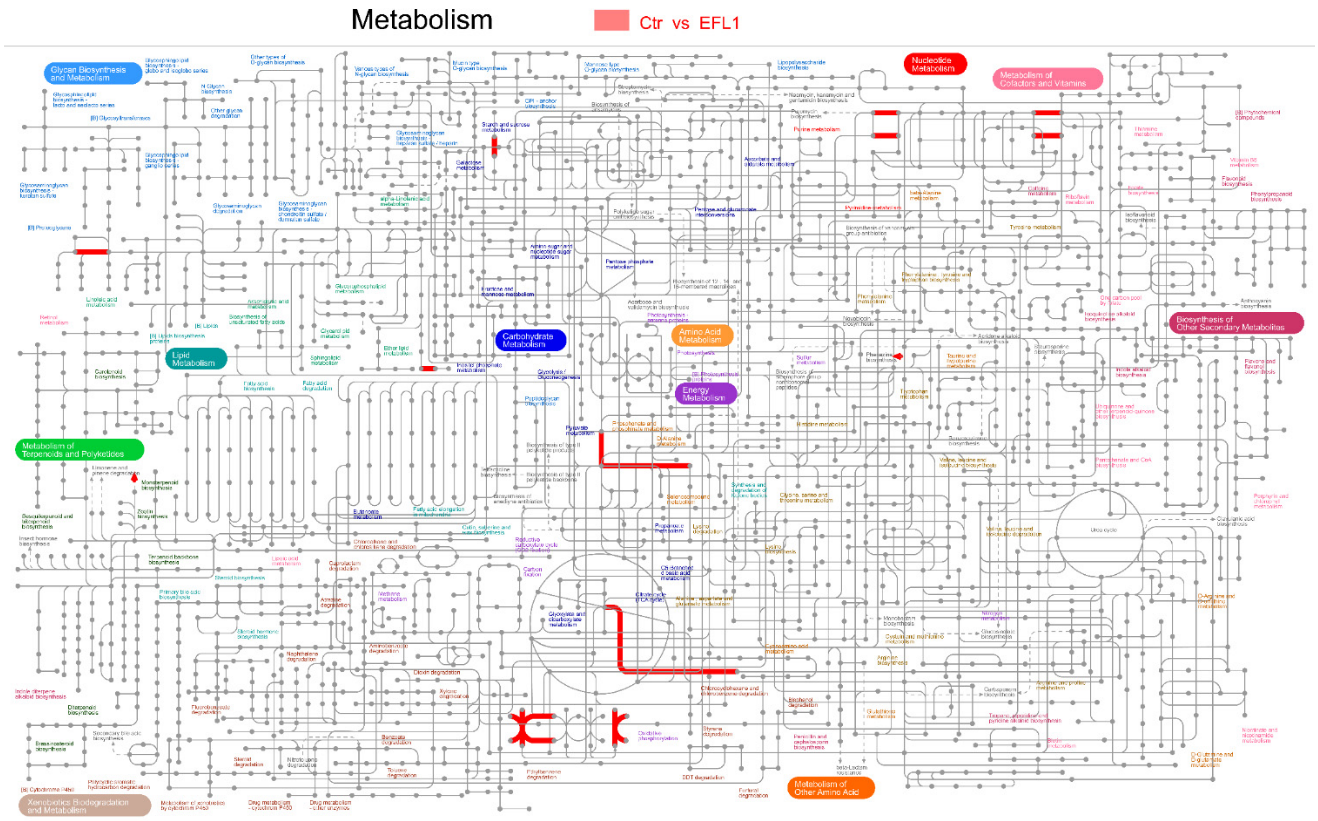

B

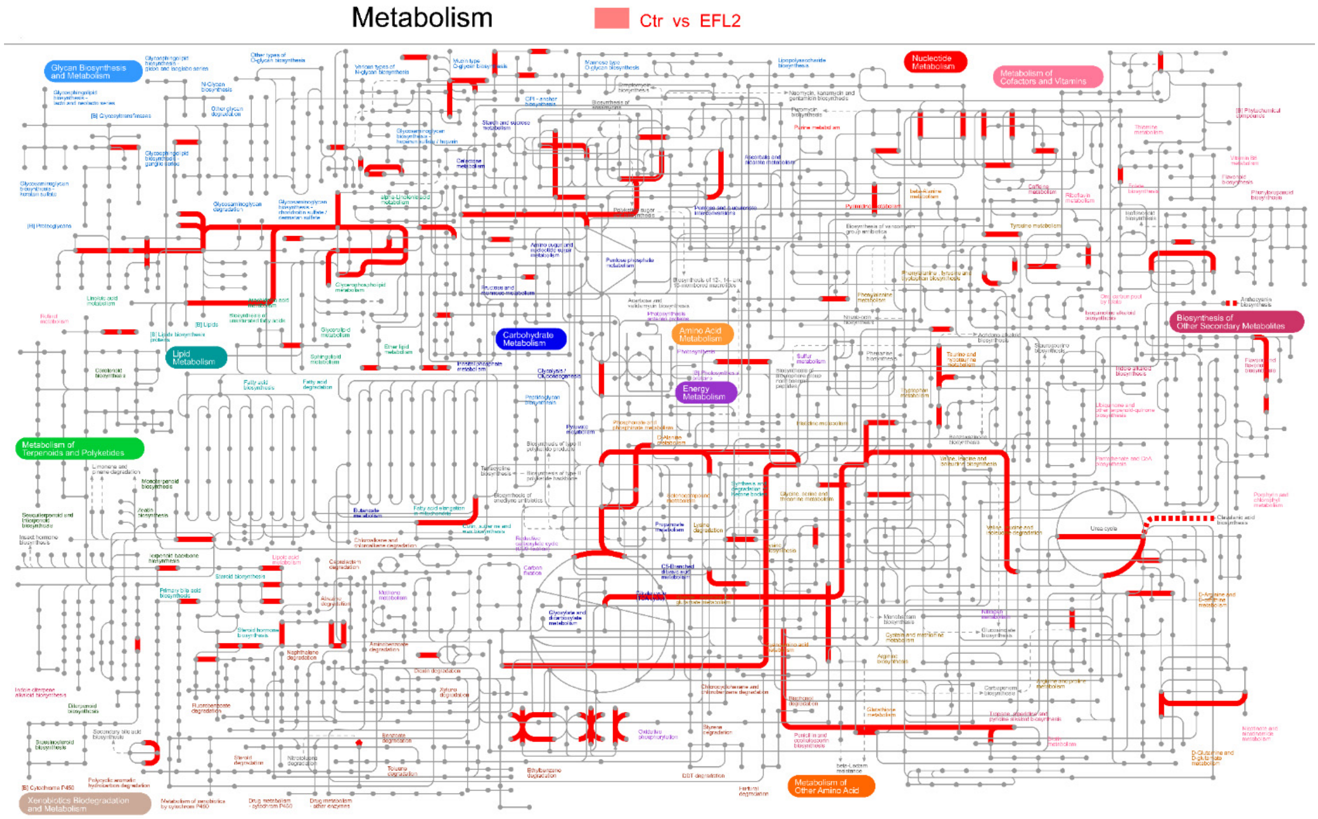

Figure S3. The visual analysis of metabolic pathways involved in differentially expressed genes after (**A**) EFL1 or (**B**) EFL2 treatment in Caco-2 cells.

Table S1. Primer sequences for miRNA of Caco-2 cells.

Gene Name	Primer Sequences (5′ to 3′)
hsa-miR-6774-3p	ATATCGTGTCCCTCTTGTCCACAG
hsa-miR-6715a-3p	TTATTATATTATTATCCAAACCAGTCGTGCCTGTGG
hsa-miR-4523	TATTATATTATTAATTAATTAATTATTATTATTATAATGACCGAGAGGGCCTCGGCTGT
hsa-miR-548av-3p	CTGTATTGCCAAAACTGCAGTTACTTTTGC
hsa-miR-616-3p	CAGTCATTGGAGGGTTTGAGCAG
hsa-miR-5090	TTATATTATTATTATTATTATTATTAATTATTCCGGGGCAGATTGGTGTAGGGTG
hsa-miR-1255b-5p	CCGGATGAGCAAAGAAAGTGGTT
hsa-miR-4284	GGGCTCACATCACCCCAT
hsa-miR-146a-3p	CGGCCTCTGAAATTCAGTTCTTCAG
hsa-miR-4458	CGGAGAGGTAGGTGTGGAAGAA

Table S2. Gene descriptions and sequences of qRT-PCR primers for mRNA of Caco-2 cells.

Genes	Gene Descriptions		Primer Sequences (5′ to 3′)
GAPDH	internal reference, encodes a member of the glyceraldehyde-3-phosphate dehydrogenase protein family	Forward	AGCCTTCTCCATGGTGGTGAA
		Reverse	ATCACCATCTTCCAGGAGCGA
MT-ND5	involves in respiratory electron transport, ATP synthesis and GABAergic synapse; affects NADH dehydrogenase (ubiquinone) activity	Forward	CTTCTAAACGCTAATCCAAGCC
		Reverse	TAGTGGGCTATTTTCTGCTAGG
ATP5G1	mitochondrial ATP synthase that catalyzes ATP synthesis and involves in H^+ transporting	Forward	GACACAGCAGCCAAGTTTATTG
		Reverse	CCAAGAATGGCATAGGAGAAGA
RPS15	encodes a ribosomal protein that is a component of the 40S subunit; further affects protein synthesis	Forward	CATGATCATCCTACCCGAGATG
		Reverse	CTTGTAGGTGATGGAGAACTCG
FADD	can be recruited by TNFRF6/Fas-receptor, and thus participates in the death signaling initiated by receptor; recruits Caspase-8; involves in early T cell development	Forward	GACCGAGCTCAAGTTCCTATG
		Reverse	CAGCATGGAGAAGAGGTCTAG
HRK	promotes apoptosis by interacting with the apoptotic inhibitors BCL-2 and BCL-X(L) via its BH3 domain	Forward	TAGGCGACGAGCTGCACCAG
		Reverse	CGCACAGCCAAGGCCAGTAG
DRD5	a G-protein coupled dopamine receptor which stimulates adenylyl cyclase; involves in dopamine-DARPP32 feedback onto cAMP pathway	Forward	CAACATGACCAACGTCTTCATC
		Reverse	CTGAGTCATCTTGCGCTTGTAG
GPR27	activation of GPR27 by neurotransmitters induces an intracellular signaling cascade mediated by heterotrimeric GTP-binding proteins or G proteins	Forward	CTCGTGCTGGAAGAATTCAAG
		Reverse	CAGAGGAGCAGGAAGAGCAG
HTR6	encodes protein stimulates AC to activate the cAMP-dependent signaling pathway; regulate cholinergic neuronal transmission in the brain	Forward	CTCTTCACGTCTGACCTGATG
		Reverse	AGCACATCACGTCGAAGG
C1orf167	involves in neural tube defects, folate-sensitive	Forward	GCGAGTGGCTCAGCAGTTACAG
		Reverse	GCTCCTCTCCTTGGGCTCCTG
ZSCAN32	involves in nucleic acid binding and DNA-binding transcription factor activity, RNA polymerase II-specific; affects gene expression and herpes simplex virus 1 infection	Forward	CTCTGAGCTACAAAAAGGCTTG
		Reverse	CACACTCTGAGAGCAGTTTTTC
MED26	regulates lipid metabolism by PPARα; involves in chromatin regulation/acetylation, and transcription coactivator activity	Forward	CAACGAGATCATCCAGTCCTAC
		Reverse	GTACTCAGACATGAAGTGGTGA
REXO1	involves in ribosome biogenesis, nucleic acid binding and exonuclease activity	Forward	CGAGATGTCCTACACCACATAT
		Reverse	CTTCACGAAGGTGTCATAAACC
UBA7	involves in ubiquitin-protein transferase activity, ubiquitin activating enzyme activity and IFN-γ signaling	Forward	GAGCTGTATAAGGTGGTGAGTG
		Reverse	GCATATAGCGGATGAGGTAGTT
SKP1	involves in TLR4 signaling activation and ubiquitin-protein transferase activity	Forward	CTGTTGCCAATATGATCAAGGG
		Reverse	GCTTCCTCCTCTTCAGTAAAGT

MT-ND5: mitochondrially encoded NADH dehydrogenase 5; **ATP5G1**: ATP synthase membrane subunit c locus 1; **RPS15**: ribosomal protein S15; **RPL7**: ribosomal protein L7; **FADD**: Fas-associating death domain-containing protein; **HRK**: harakiri, BCL2 interacting protein; **DRD5**: dopamine receptor D5; **DARPP32**: protein phosphatase 1 regulatory inhibitor subunit 1B; **cAMP**: cyclic adenosine monophosphate; **GPR27**: G protein-coupled receptor 27; **GPCR**: G protein-coupled receptors; **GTP**: guanosine triphosphate; **HTR6**: 5-hydroxytryptamine receptor 6; **AC**: adenylate cyclase; **C1orf167**: chromosome 1 open reading frame 167; **ZSCAN32**: zinc finger and SCAN domain containing 32; **MED26**: mediator complex subunit 26; **PPAR**α: peroxisome proliferator-activated receptor α; **REXO1**: RNA exonuclease 1 homolog; **UBA7**: ubiquitin like modifier activating enzyme 7; **IFN-**γ: interferon gamma; **SKP1**: S-phase kinase associated protein 1; **TLR4**: toll like receptor 4.

Table S3. Summary of reads and mapping rate of mRNA sequences of Caco-2 cells with or without EFLs treatment.

Sample	Raw reads	Raw bases	Clean reads	Clean bases	Error rate (%)	Q20 (%)	Q30 (%)	GC content (%)	Total mapped	Multiple mapped	Uniquely mapped
Ctr-1	69,236,184	10,454,663,784	68,345,726	10,182,603,020	0.0246	98.28	94.49	51.16	66,781,609 (97.71%)	5,220,604 (7.64%)	61,561,005 (90.07%)
Ctr-2	67,538,362	10,198,292,662	66,651,606	9,892,097,424	0.0247	98.23	94.40	50.26	64,818,094 (97.25%)	4,813,283 (7.22%)	60,004,811 (90.03%)
Ctr-3	71,452,610	10,789,344,110	70,345,602	10,460,289,224	0.0249	98.13	94.13	50.65	68,533,822 (97.42%)	5,210,079 (7.41%)	63,323,743 (90.02%)
EFL1-1	71,675,282	10,822,967,582	70,696,056	10,520,449,792	0.0246	98.27	94.44	50.29	69,021,488 (97.63%)	5,172,210 (7.32%)	63,849,278 (90.32%)
EFL1-2	78,465,984	11,848,363,584	77,327,154	11,435,726,413	0.0244	98.32	94.61	51.05	75,110,013 (97.13%)	6,241,215 (8.07%)	68,868,798 (89.06%)
EFL1-3	73,848,068	11,151,058,268	72,731,590	10,765,344,434	0.0243	98.36	94.71	51.04	71,016,067 (97.64%)	5,666,352 (7.79%)	65,349,715 (89.85%)
EFL2-1	78,039,270	11,783,929,770	77,017,708	11,428,915,879	0.0247	98.23	94.38	51.02	75,147,803 (97.57%)	5,980,293 (7.76%)	69,167,510 (89.81%)
EFL2-2	74,194,114	11,203,311,214	73,208,196	10,895,787,620	0.0245	98.32	94.60	51.38	71,213,395 (97.28%)	5,799,621 (7.92%)	65,413,774 (89.35%)
EFL2-3	60,877,848	9,192,555,048	60,192,712	8,935,790,134	0.0245	98.30	94.53	50.69	58,133,368 (96.58%)	4,771,234 (7.93%)	53,362,134 (88.65%)

Q20 and Q30 refers to the percentage of nucleotides with Phred quality score >20 and >30, respectively.

Table S4. Summary of reads and mapping rate of miRNA sequences of Caco-2 cells with or without EFLs treatment.

Sample	Raw reads	Raw bases	Clean reads	Clean bases	Q20 (%)	Q30 (%)	GC content (%)	Useful reads (18nt-32nt)	Mapped reads	Mapped reads (+)	Mapped reads (-)
Ctr-1	17,060,405	1,279,530,375	14,999,942	366,748,644	99.38	98.11	49.63	12,219,317	8,437,420	5,854,171	3,882,299
Ctr-2	19,638,714	1,472,903,550	17,318,550	411,219,525	99.42	98.20	50.96	13,912,392	8,292,228	5,872,253	3,699,549
Ctr-3	21,160,021	1,587,001,575	18,298,666	426,286,469	99.46	98.33	51.10	14,535,827	8,623,844	6,082,484	3,920,111
EFL1-1	19,298,799	1,447,409,925	16,296,066	384,036,281	99.43	98.25	49.20	13,408,350	9,516,724	6,546,403	4,440,699
EFL1-2	18,769,439	1,407,707,925	16,130,418	377,127,130	99.44	98.26	51.96	12,299,154	6,794,079	4,831,308	3,074,336
EFL1-3	16,885,751	1,266,431,325	13,760,541	307,709,657	99.42	98.21	52.21	10,281,867	5,625,063	4,020,570	2,538,832
EFL2-1	18,521,264	1,389,094,800	16,307,924	376,572,879	99.45	98.28	48.25	13,960,541	10,420,152	7,239,002	4,795,640
EFL2-2	17,951,900	1,346,392,500	15,546,882	360,572,577	99.44	98.25	51.58	12,001,510	6,597,082	4,635,835	3,099,833
EFL2-3	20,319,301	1,523,947,575	18,063,748	436,360,344	99.44	98.25	51.79	13,610,244	7,504,044	5,257,983	3,553,943

Mapped reads (+) and mapped reads(-) are the reads located in the positive- and negative-strand RNA, respectively.

Table S5. Functional annotation of transcriptome data in six public databases.

Data base	Transcript number (percent)	Gene number (percent)
COG	174,005 (83.44%)	40,502 (68.78%)
GO	14,767 (70.57%)	31,485 (53.47%)
KEGG	140,001 (67.14%)	28,351 (48.15%)
NR	186,400 (89.39%)	45,614 (77.46%)
Swiss-Prot	171,322 (82.16%)	39,390 (66.89%)
Pfam	85,855 (41.17%)	16,553 (28.11%)
Total annotation	203,268 (97.48%)	46,195 (78.45%)
Total	208,527 (100.00%)	58,884 (100.00%)

Table S6. The miRNA numbers including known and novel miRNAs in each sample.

Sample	Known miRNAs	Novel miRNAs	Total
Ctr-1	1185	338	1523
Ctr-2	1135	357	1492
Ctr-3	1140	374	1514
EFL1-1	1197	364	1561
EFL1-2	1086	350	1436
EFL1-3	1044	350	1394
EFL2-1	1189	346	1535
EFL2-2	1091	361	1452
EFL2-3	1109	366	1475

Table S7. The AS number in Caco-2 cells treated with EFLs.

AS types	Ctr VS EFL1			Ctr VS EFL2		
	Exon exclusion (ΔPSI<0)	Exon inclusion (ΔPSI>0)	Total events	Exon exclusion (ΔPSI<0)	Exon inclusion (ΔPSI>0)	Total events
SE	862	637	1499	1772	2058	3830
MXE	74	89	163	220	282	502
A5SS	63	59	122	180	139	319
A3SS	68	70	138	180	160	340
RI	58	42	100	303	91	394
Total	1125	897	2022	2655	2730	5385

SE, skipped exon; A5SS, alternative 5'splice site; A3SS, alternative 3'splice site; MXE, mutually exclusion exon; RI, retained intron. PSI (percent spliced in) = splice in / (splice in + splice out), ΔPSI (Ctr/EFL) = PSI (Ctr) — PSI (EFL), ΔPSI<0 means the percentage of exon exclusion in Ctr group is higher than EFL group, and ΔPSI>0 means the percentage of exon inclusion in Ctr group is higher than EFL group.

Table S8. The Indel regions distribution after EFLs treatment in Caco-2 cells.

Indel regions	Ctr	EFL1	EFL2
splicing	3.3±0.6	2.7±0.6	2.3±0.6
upstream-downstream	8.0±0.0	9.0±2.0	8.3±0.6
downstream	68.0±2.0	68.3±4.0	65.7±4.2
exonic-splicing	N.D.	N.D.	N.D.
UTR5	74.7±2.1	75.3±0.6	74.3±3.1
ncRNA_exonic	182.3±6.1	185.7±2.1	185.7±7.6
intergenic	386.3±17.6	367.3±34.3	343.3±12.9
ncRNA_UTR5	3.3±0.6	2.7±0.6	3.0±1.0
intronic	1163.7±35.3	1110.7±51.0	970.7±83.9
ncRNA_UTR3	71.3±1.5	70.0±2.0	70.0±2.0
ncRNA_splicing	N.D.	N.D.	N.D.
UTR3	862.3±6.8	856.7±4.2	865.3±5.9
exonic	71.3±2.1	73.0±0.0	72.7±2.5
upstream	45.0±5.6	46.3±4.9	43.3±7.4
UTR5-UTR3	3.0±0.0	3.0±0.0	3.0±0.0
ncRNA_UTR5-ncRNA_UTR3	1.0±0.0	1.0±0.0	1.0±0.0
ncRNA_intronic	296.7±7.6	285.7±16.0	280.0±40.1
Total	3240.3±17.9	3157.3±101.0	2988.7±149.5*

*P < 0.05 compared with vehicle control.

Table S9. The SNP number in Caco-2 cells treated with EFLs.

SNP types	Ctr	EFL1	EFL2
Transition			
A/G	154,626.7±1605.4	154,189.2±4022.0	139,284.3±9366.7
C/T	68,788.0±891.7	68,233.3±2387.9	62,093.7±5116.0*
G/A	68,623.7±683.7	68,256.7±2480.0	61,889.0±5126.2*
T/C	151,934.3±1969.0	151,678.7±4095.7	137,447.3±8923.9
Transversion			
A/C	13,052.7±275.5	12,997.0±566.0	11,756.7±1059.0
A/T	13,222.3±266.7	13,065.3±538.5	11,866.3±1077.7
G/C	17,440.7±236.1	17,402.7±685.4	15,866.7±1313.8
G/T	16,598.7±105.9	16,522.7±615.3	15,006.0±1231.3
C/A	16,484.7±112.3	16,460.7±657.4	15,002.0±1181.0
C/G	17,364.3±249.7	17,273.0±607.7	15,794.3±1317.2
T/A	13,129.7±342.7	13,012.0±555.2	11,687.0±1045.8*
T/G	13,183.3±167.1	13,047.7±563.7	11,870.7±976.2
Total	564,449.0±6755.3	562,139.0±17,228.9	509,564.0±37716.7*

*$P < 0.05$ compared with vehicle control.

Table S10. The SNP regions distribution after EFLs treatment in Caco-2 cells.

SNP regions	Ctr	EFL1	EFL2
splicing	2686.3±124.5	2722.7±43.1	2423.0±199.4
upstream-downstream	754.3±35.0	773.3±41.2	758.0±21.0
downstream	8218.7±120.5	8288.3±231.8	8270.3±374.0
exonic-splicing	122.3±6.1	121.0±2.0	119.3±11.0
UTR5	7137.3±145.1	7217.3±127.6	7102.0±338.9
ncRNA_exonic	24,029.7±277.6	24,129.3±386.6	23,435.7±1017.3
intergenic	62,057.3±1027.0	62,413.7±2699.4	58,713.7±4695.3
ncRNA_UTR5	488.3±11.0	488.0±9.5	483.7±22.0
intronic	327,199.0±9177.1	324,927.7±11,319.6	282,375.0±24,812.4
ncRNA_UTR3	3539.67±43.25	3543.67±6.51	3575.0±42.6
ncRNA_splicing	97.0±6.6	101.7±9.2	92.0±5.3
UTR3	41,044.0±173.2	41,266.7±381.0	41,265.3±341.1
exonic	22,478.3±195.6	22,454.0±129.3	22,424.3±521.5
upstream	5575.3±364.0	5481.3±261.5	5369.7±450.0
UTR5-UTR3	147.0±1.0	149.7±2.1	148.3±4.9
ncRNA_UTR5-ncRNA_UTR3	5.0±1.0	5.3±1.5	7.0±1.0
ncRNA_intronic	58,869.3±660.0	58,055.3±2782.7	53,001.7±4946.9
Total	564,449.0±6755.3	562,139.0±17,228.9	509,564.0±37,716.7*

*$P < 0.05$ compared with vehicle control.
